# A new patient with a terminal *de novo* 2p25.3 deletion of 1.9 Mb associated with early-onset of obesity, intellectual disabilities and hyperkinetic disorder

**DOI:** 10.1186/1755-8166-7-53

**Published:** 2014-08-05

**Authors:** Maria Clara Bonaglia, Roberto Giorda, Sergio Zanini

**Affiliations:** 1 Cytogenetics Laboratory, Scientific Institute, IRCCS Eugenio Medea, Via Don Luigi Monza, 20, 23842 Bosisio Parini, Lecco, Italy; 2 Molecular Biology Laboratory, Scientific Institute, IRCCS Eugenio Medea, Bosisio Parini, Lecco, Italy; 3 Unit for Severe Disabilities in Developmental Age, Scientific Insitute, IRCCs Eugenio Medea, Udine, Italy

**Keywords:** Deletion 2p25.3, Obesity, Language delay, Hypekinetic disorder, *MYT1L*

## Abstract

Terminal and interstitial deletions of 2p25.3 (size < Mb), detected by array-CGH analysis, have been reported in about 18 patients sharing common clinical features represented by early-onset obesity/ overweightness associated with intellectual disabilities (ID) and behavioural troubles. This observations led to hypothesize that 2p subtelomeric deletion should be associated with syndromic obesity and *MYT1L* became the main candidate gene for ID and obesity since it is deleted or disrupted in all hitherto published cases.

Here we described a 2p25.3 *de novo* terminal deletion of 1.9 Mb, of paternal origin, detected by array-CGH analysis in a girl of 4.4 years with a distinctive phenotype consisting of early-onset of obesity associated with moderate ID, and hyperkinetic disorder. The deletion disrupted *MYT1L* and encompassed five other OMIM genes, *ACP1, TMEM18, SNTG2, TPO,* and *PXDN.*

Here, we discuss the combined functional effects of additional haploinsufficient genes, that may concur with heterozygous deletion of *MYT1L*, in the aetiology for syndromic obesity associated with 2p25.5 subtelomeric deletion.

## Background

Monosomies of 2p are very rare and are usually observed in more complex aberrations such as inverted/duplications [1], ring 2 chromosomes [2-4], or a derivative chromosome 2 ([5] one case) making a definite correlation between severity of the phenotype and size or type of the aberrations still difficult.

Recently, terminal and interstitial deletions of 2p25.3 ranging in size from 0.37 Mb to 3.11 Mb, detected by genome-wide array analysis, led to the definition of the smallest region of overlap (SRO) for pure 2p25.3 deletions in patients with intellectual disabilities (ID) [6] and early-onset obesity [5]. This region harbours the myelin transcription factor 1-like gene (*MYT1L,* OMIM: 613084), coding for a neural transcription factor with a pivotal role in regulating neuron differentiation [7,8], a good candidate gene for ID in patients with pure 2p25.3 deletion [6] and early-onset obesity [5].

Here we report a new patient with moderate intellectual disability (ID), early-onset obesity, and hyperkinetic syndrome associated to a 2p25.3 simple *de novo* terminal deletion of 1.9 Mb disrupting *MYT1L.*

The causative role of *MYT1L* haploinsufficiency in 2p25.3 deletion and the involvement of 2pter monosomy in the chromosomal aetiology for syndromic obesity are discussed.

## Case presentation

**Figure 1:**
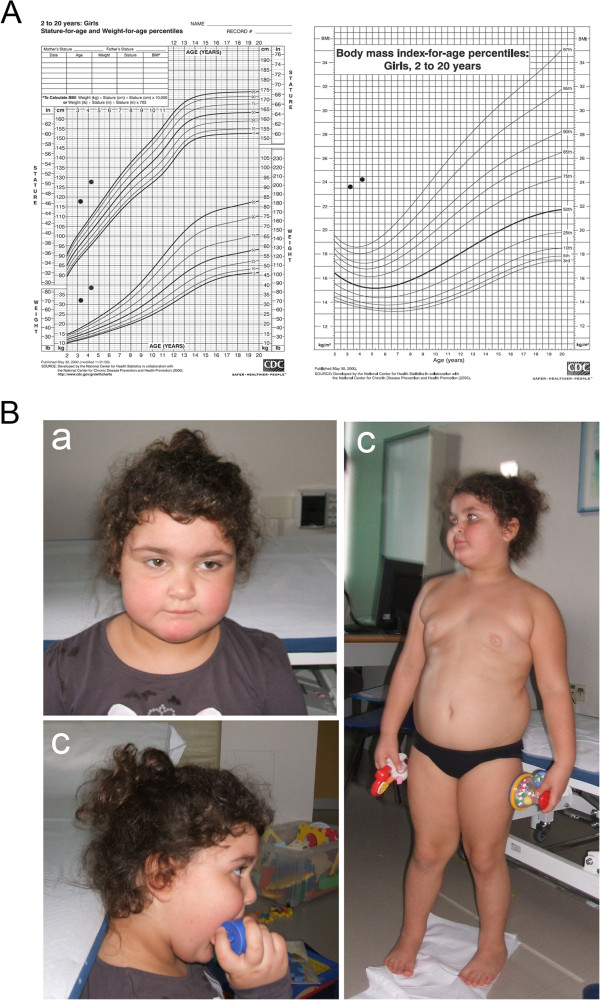
**Details of growth - BMI-for age and facial/physical appearance of the patient. A**. On the left, growth curves for our patient showing early-onset obesity; on the right, BMI at 41 and 53 months. The source of growth and BMI-for-age graphic is: http://www.chartsgraphsdiagrams.com/HealthCharts/growth-2-20-girls.html. **B**. Photographs of the patient: (a) frontal face, (b) lateral face, and (c) full frontal views. Note the square-shaped truncal build.

Informed consent was obtained from both parents of the patient.Frontal and lateral view of the patient as well as growth curves are shown in Figure 1.

The patient came to our attention at the age of 3 years for intellectual disabilities, obesity, macrosomy, and hyperkinetic syndrome of unknown aetiology. She is a female, the only child of healthy non-consanguineous 39-year-old mother and 39-year-old father with unremarkable family history. The father and mother were of normal height and weight. She was born at 41 weeks of gestation by caesarean section, due to spontaneous amniotic sac rupture without uterine contractions. Birth parameters were in range (weight: 3090 gr, length: 50.5 cm, cranial circumference-OFC: 32 cm). Apgar scores were 9/10 at ′/5′, respectively. The perinatal period was unremarkable with good neonatal adaptation to extra uterine environment and breast feeding; neither hypotonia nor jaundice were present. Her psychomotor development was characterised by sitting unsupported at 15 months, walking autonomously at 18 months, and uttering her first words at 18 months. The child developed obesity during early infancy, despite normal food-seeking behaviour (Additional file 1: Table S1.) and on our first examination at the age of 41 months her weight was 32 Kg (BMI = 23.59) (Figure 1A), length 128 cm ( > >97^th^c), OFC 51 cm (50-75^th^c). At the age of 3 years, she showed mild to moderate intellectual disabilities (Leiter-R scale IQ = 79) with main deficit in the verbal area, both in language comprehension and production. She was able to produce less than 10 words and unable to construct even the simplest sentences. The Vineland Adaptive Behaviour Scale (VABS) was completed by interviewing both parents. Patient’s age equivalent (AE) was less than 1 year 9 months; receptive language and expressive skills score was 1 year 7 months; socialization AE was less than 1 year 11 months; daily living skills AE was 2 years 2 months; motor skills AE was 2 years 9 months.

Asynergistic walking with sole-sole pattern with feet intra-rotation due to intra-rotation of the tibia bilaterally and anteroversion of the femurs’ neck has been observed.

At ocular evaluation, bilateral amblyopia (4/10) with mixed astigmatism was observed. Audiometric examination and brain-stem auditory evoked potentials resulted within normal range, with the exception of a mild delay of the 1st wave in the right ear due to mild transmission deficits.

Electroencephalograms (EEG) recording data, acquired during wake and spontaneous sleep, revealed epileptic anomalies only during profound sleep.

At last evaluation, at the age of 4 y and 4 mo, her growth parameters were: weight 39 kg (> > 97^th^c), length 128 cm (> > 97^th^c), OFC 51 cm (50-75^th^c), BMI 24.18 (Figure 1A). Facial examination showed slightly upturned nose and short neck without other remarkable dimorphisms (Figure 1B). Her behaviour was mainly characterised by a severe form of hyperkinetic disorder. Her attention dwelt no more than a few minutes on a single information or activity, even when she was extremely interested and highly focused on it by the examiner’s intervention. Her spontaneous play activities are basically unstructured: she holds toys, explores them rapidly and superficially, then beats them. She appreciates sensory-motor experiences in play. She has no play strategies. Spontaneous interaction with relatives, other adults, and peers is poor but present. At first glance, she appears to be an autistic girl. However, when faced by the examiner and stimulated by being presented toys, she spontaneously tries to share her activities and eye contact is present. She attends kindergarten for normally developing children; however, she has a support teacher for the whole period of her school attendance. She also has a special teaching schedule and program. At present, her cognitive functioning is equivalent to moderate mental retardation with hyperkinetic syndrome. At last evaluation EEG, compared with the previous registration, is remarkably better due to a significant reduction of non-convulsive paroxysmal activity during profound sleep.

Brain MRI showed increased subdural peri-encephalic space in the frontal regions, and enlarged cisterna magna without signs of compression on the 4^th^ ventricle or the cerebellar vermis. No other remarkable signs were evident. At abdominal echography, no pathological signs concerning spleen, liver, gallbladder, pancreas, kidneys, or bladder were observed.

Electrocardiogram (ECG) showed regular sinusoidal rhythm with 106 bites per minute, conduction and repolarization within normal limits, QT tract corrected 385 msec, and arterial hypertension; no pathological signs were evident at heart echography.

Routine haematological exams revealed increased levels of platelets (420,000 – normal range 150,000-400,000) increased triglycerides (217 mg/dL – normal range 40–150) with normal total cholesterol (143 mg/dl- normal range 120–140). Thyroid function was normal (TSH: 1.198 Î¼UI/ml (N:0.350-5000), T4:13.4 pg/ml (N:8.9-17.6), and T3: 4.8 pg/ml (N:2.3-4.2)).

**Figure 2:**
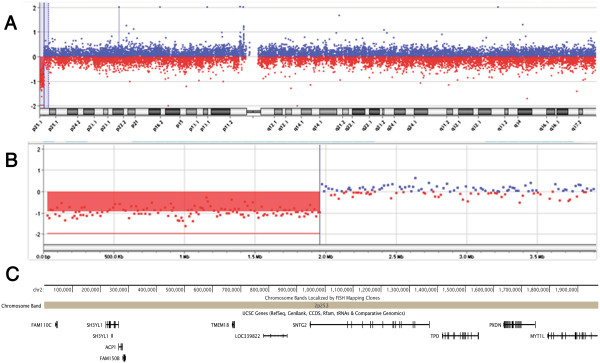
**Molecular details of 2p25.3 deletion.***Array- CGH profile of chromosome 2* showing **(A)** a terminal deletion of 1.9 Mb at 2p25.3, and **(B)** an enlargement of the 2p25.3 deletion. *Genomic view of the terminal 1.9 Mb of chromosome 2p25.3***(C)**: UCSC genes (GRCh37/hg19) are shown. The screenshot shows the deletion breakpoint disrupting *MYT1L.*

Array-CGH analysis, performed with an Agilent Human Genome CGH Microarray Kit 180 k (Agilent Technologies Inc., Santa Clara, CA) with a resolution of ~40 Kb revealed a deletion of 1.9 Mb in chromosomal band 2p25.3 (Figure 2A) including the following genes : *FAM110C* (OMIM *611395) *ACP1* (OMIM *171500), *TMEM18* (OMIM *613200), *SNTG2* (OMIM *608715), *TPO* (OMIM *608715), *MYT1L* (OMIM *613084) *PXDN* (OMIM *605158) and *SH3YL1*, *FAM150B* (Figure 2B). All nucleotide positions refer to the Human Genome, Feb 2009 Assembly (hg19). Data analysis was performed using Agilent Cytogenomics version 2.5.8.1. The deletion breakpoint, located between 1,957,657 Mb and 1,973,115 Mb (hg19), lies within intron 6 of *MYT1L*.

Real-time quantitative PCR (qPCR) assays, performed on the patient and her parents using SYBR Green and analysed on an ABI PRISM 7900HT sequence detection system (Applied Biosystems, Foster City, CA), demonstrated that the 2p25.3 deletion originated *de novo* (data not shown).

Genotyping of polymorphic locus D2S2268 in the proband and her parents, performed by amplification with primers labelled with fluorescent probes (ABI 6-Fam and 8-Hex) followed by analysis on ABI 3500AV Genetic Analyser (Applied Biosystems), revealed the paternal origin of the deletion (data not shown). The final interpretation of the rearrangement was arr[hg19] 2p25.3(30,341-1,957,657x1,1,973,115x2) dn.

Including the present case, about 18 patients, with ages ranging from infancy to adulthood and partially overlapping 2p25.3 deletions, have been described so far [5,6]. All these patients share common distinctive clinical features represented by early-onset obesity/ overweightness associated with ID and behavioural troubles [5,6]. The first symptom observed in our patient was very early-onset obesity (Figure 1A), starting from the 3^rd^ month of life (Additional file 1: Table S1). A square-shaped truncal build was evident (Figure 2B), as previously reported in four patients out of six by Steven et al. [6] and two out of five by Doco-Fenzy et al. [5]. Her psychomotor development, resulting in mild to moderate ID, was mainly characterized by severe language delay (less than 10 words) and inability to produce sentences, even the simplest ones.

By combining genotype-phenotype analysis from all hitherto reported patients and DECIPHER cases, *MYT1L* has recently been proposed as the major candidate gene for ID [6] and early-onset obesity [5], since the gene is deleted in all patients with terminal or interstitial monosomy of 2p25.3 [5,6]. The causative role of *MYT1L* haploinsufficiency for early-onset obesity is further reinforced by our finding, showing that our patient’s proximal deletion breakpoint lies within a segment of 15,459 bp located in the sixth intron of *MYT1L* (Figure 2C). A very similar 2p25.3 deletion of 1.975 Mb with its deletion breakpoint within intron 5 of *MYT1L* has been recently reported in another patient (PZ1 from [5]) with clinical features overlapping those of our patient, apart from neonatal hypotonia and failure to thrive. As hypothesized by Doco-Fenzy et al. [5], *ACP1* and *TEM18M*, usually deleted in the majority of patients with obesity or overweight, may concur to the early-onset obesity/overweight observed in patients with 2pter deletions. We may thus hypothesise that haploinsufficiency of *ACP1*, a gene associated with severe obesity and increased total cholesterol and triglycerides levels [9,10], may also be responsible for the increased levels of triglycerides and obesity observed in our patient.

Patients with small interstitial deletion involving *MYT1L* (P5, SP6 and ID25513 from [5]) manifest behavioural troubles, including autistic spectrum disorders (ASD) while subjects with 2p25.3 terminal deletions additionally involving *SNTG2* (P1-2,P4 , SP5-6 from [5]) manifest aggressiveness and outbursts [5,6,11].

Our patient carrying a 2p25.3 deletion spanning nine genes, including *SNTG2* and *MYT1L* (Figure 2B) manifests neither ASD nor aggressiveness and outburst. Her spontaneous interaction with relatives, other adults, and peers is poor but present and, if stimulated, she spontaneously tries to share her play activities. Her behaviour is mainly characterised by a severe form of hyperkinetic disorder. Overall, hyperactivity appears to be the main behavioural trait observed in patients with 2p25.3 monosomy.

## Conclusions

In conclusion, we identified a *de novo* 1.9 Mb deletion of 2p25.3 in a girl with distinctive phenotype characterised by early-onset obesity, ID, and hyperkinetic disorder. The deletion breakpoint disrupting the *MYT1L* gene reinforces the hypothesis of its primary causative role in ID, hyperactivity, and obesity.

*MYT1L* is disrupted or entirely deleted in all hitherto reported patients with subtelomeric 2pter microdeletions. Combined functional effects of additional multiple haploinsufficient genes, usually deleted in patients with 2p25.3 monosomy, may concur to cause the observed clinical signs. Our data further reinforce the notion that subtelomeric 2p25.3 deletion (size < Mb) results in syndromic obesity.

## Consent

Written informed consent was obtained from the parents of the patient for publication of this Case Report and any accompanying images. A copy of the written consent is available for review by the Editor-in-Chief of this journal.

## Competing interests

The authors declare that they have no competing interests.

## Authors’ contributions

MCB performed array-CGH analysis and wrote the paper; RG performed qPCR and microsatellite analysis; SZ was responsible for the patient’s clinical follow-up examination and contributed to the clinical description. All authors read and approved the final manuscript.

## Supplementary Material

Additional file 1: Table S1 Growth curves of the patient. Click here for file
