# Supplementary material for: A new patient with a terminal de novo 2p25.3 deletion of 1.9 Mb associated with early-onset of obesity, intellectual disabilities and hyperkinetic disorder
Source: Mol Cytogenet. 2014 Aug 5;7:53. doi: 10.1186/1755-8166-7-53 (PMC4131807; doi:10.1186/1755-8166-7-53)
Supplement: Additional file 1: Table S1 — Growth curves of the patient. [file 1755-8166-7-53-S1.docx]

**Additional file 1: Table S1. Growth curves of the patient**

|  | **Weight** | **Lenght** | **Cranial circumference-OFC** |
| --- | --- | --- | --- |
| At birth | 3.09 Kg (50°C) | 50.5 cm  (50-75°C) | 32 cm  (3-5°C) |
| 1^st^ month | 4.5 Kg (75°C) | 57 cm  (90°C) | 35.5 cm  (10°C) |
| 3^rd^ month | 6.2 Kg  (75-90°C) | 64 cm (>97°C) | 39.8 cm  (50°C) |
| 4^th^ month | 7 Kg  (90°C) | 68 cm (>97°C) | 41 cm  (50°C) |
| 8^th^ month | 10.5 Kg (>97°C) | 76 cm (>97°C) | 41.4 cm  (3-10°C) |
| 12^th^ month | 11.5 Kg (90-97°C) | 85 cm (>97°C) | 43.5 cm (10°C) |
| 19^th^ month | 16 Kg (>97°C) | 92 cm (>97°C) | 44.8 cm  (10°C) |
| 24^th^ month | 18 Kg (>>97°C) | 102 cm (>>97°C) | 45.8 cm  (10°C) |
| 41^st^ month | 32 Kg (>>97°C) | 128 cm (>>97°C) | 51 cm (50-75°C) |
